# Evidence for Impaired CARD15 Signalling in Crohn's Disease without Disease Linked Variants

**DOI:** 10.1371/journal.pone.0007794

**Published:** 2009-11-12

**Authors:** Jakob Benedict Seidelin, Oliver Jay Broom, Jørgen Olsen, Ole Haagen Nielsen

**Affiliations:** 1 Department of Gastroenterology, Medical Section, Herlev Hospital, University of Copenhagen, Denmark; 2 Institute of Cellular and Molecular Medicine, The Panum Institute, University of Copenhagen, Denmark; Charité-Universitätsmedizin Berlin, Germany

## Abstract

**Background:**

Sensing of muramyl dipeptide (MDP) is impaired in Crohn's disease (CD) patients with disease-linked variants of the CARD15 (caspase activation and recruitment domain 15) gene. Animal studies suggest that normal CARD15 signalling prevents inflammatory bowel disease, and may be important for disease development in CD. However, only a small fraction of CD patients carry the disease linked CARD15 variants. The aim of this study was thus to investigate if changes could be found in CARD15 signalling in patients without disease associated CARD15 variants.

**Methodology/Principal Findings:**

By mapping the response to MDP in peripheral monocytes obtained from CD patients in remission not receiving immunosuppresives, an impaired response to MDP was found in patients without disease linked CARD15 variants compared to control monocytes. This impairment was accompanied by a decreased activation of IκB kinase α/β (IKKα/β), the initial step in the nuclear factor κB (NFκB) pathway, whereas activation of mitogen-activated protein (MAP)-kinases was unaffected. MDP additionally stimulates the inflammasome which is of importance for processing of cytokines. The inflammasome was constitutively activated in CD, but unresponsive to MDP both in CD and control monocytes.

**Conclusions/Significance:**

These results suggest that inhibited MDP-dependent pathways in CD patients not carrying the disease-associated CARD15 variants might be of importance for the pathogenesis of CD. The results reveal a dysfunctional immune response in CD patients, not able to sense relevant stimuli on the one hand, and on the other hand possessing constitutively active cytokine processing.

## Introduction

There is growing evidence of an impaired innate inflammatory response playing a key role in the pathogenesis of Crohn's disease (CD) [1]. The innate immune system is based on the ability to recognise pathogen-associated molecular patterns (PAMPs), like flaggelin, CpG DNA, double stranded RNA and bacterial cell wall constituents. The PAMPs are recognised by pattern recognition receptors (PRRs) located both on the cell surface (the Toll-like receptors), as well as intracellularly (the NOD-like receptors (NLRs)) [2].

Three common variants of the NLR gene caspase activation and recruitment domain 15 (CARD15, also known as nucleotide binding and oligomerisation domain 2 (NOD2)) has been associated with CD: SNP 8, 12 and 13 [3], [4]. CARD15 recognises the PAMP muramyl dipeptide (MDP), which is a peptidoglycan constituent of the cell wall of both gram-negative and gram-positive bacteria and is the minimal motif recognised by CARD15 [5]. Interaction between MDP and CARD15 leads to activation of the nuclear factor κB (NFκB) by binding of the adaptor protein RIP2 to CARD15 via caspase recruitment domains (CARDs) on both proteins [5], [6]. RIP2 activates NFκB both by down-regulation of the NFκB inhibitor, IκBα, and by activation of the TAK1 kinase and the IκB Kinase (IKK) complex [7]. NFκB subsequently translocates into the nucleus, where it acts as a transcription factor for pro-inflammatory mediators, including the cytokines tumour necrosis factor-α (TNF-α) and interleukin-1β (IL-1β) [6]. CARD15 stimulation also leads to activation of the mitogen-activated protein (MAP) kinases, p38 and JNK, through binding of CARD9 to CARD15 [8]. TAK1 has also been proposed to be an upstream activator of MAP kinases [7].

MDP is also able to activate a pro-inflammatory response directly, i.e. by activating the inflammasome, which in turn activates the pro-inflammatory caspase 1 enzyme that cleaves the pro-inflammatory cytokines IL-1β and IL-18 leading to their activation [9]. MDP bind to NALP3 thereby facilitating binding to ASC (apoptosis associated speck-like protein containing a CARD) by a pyrin domain (PYD)-PYD interaction. ASC contains a CARD domain that recruits pro-caspase 1, which is subsequently cleaved and activated [10]. NALP1 has recently been shown to possess a similar ability to sense MDP directly and interestingly MDP activated CARD15 also activates NALP1 [11], [12]. Activated caspase 1 and IL-1β has been shown to be co-secreted into the extracellular space [13].

The role of CARD15 in the pathogenesis of CD is still debated. A number of studies have shown that the CARD15 variants associated with CD cause impaired NFκB activation of peripheral mononuclear cells [14]–[16]. However, other studies have shown a gain-of-function phenotype for these mutations in monocytes isolated from CARD15 mutated mice [17]. A recent study has shown that monocytes from patients with the CARD15 mutations have a decreased negative regulation of cytokine production caused by high doses of MDP, consistent with a gain-of-function phenotype [18]. The majority of studies on human cells, however, point to a decreased inflammatory response to PAMPs in common CD-associated CARD15 variants. Curiously, the studies that have shown an impaired NFκB activation did not report direct effects of CARD15 stimulation by MDP: Differences between non-mutated and mutated CARD15 monocytes were observed in experiments employing co-stimulation with MDP and PAMPs known to stimulate PRRs other than CARD15 [14], [18], [19]. This suggests crosstalk between different PRR pathways. Mutated CARD15 alleles are, however, only found in up to one fourth of the patients with CD, which indicates that other mechanisms must be of importance in the monocyte dysfunction [20].

The above-mentioned studies have mostly been performed on unseparated circulating mononuclear blood cells, and the effects of stimulation have been determined on cytokine production[14]–[16], [19]. Increased cytokine production in cells stimulated by PAMPs could be caused by both activation of transcription dependent (i.e. CARD15 dependent) pathways or via direct stimulation of cytokine processing (i.e. inflammasome/CARD15 dependent) or a combination of these. Although we have previously reported that pro-inflammatory caspases are upregulated in ulcerative colitis [21], the role of the inflammasome activation in CD disease remains unclear.

The aim of the present study was to determine the pattern of response to MDP in CARD15 non-mutated monocytes from patients with CD compared with CARD15 non-mutated monocytes from control subjects. Both CARD15 and inflammasome responses to pure MDP stimulations were determined in order to elucidate whether alterations other than to CARD15, could be involved in the pathogenesis of CD. Since MDP activates both CARD15 pathways and the inflammasome, it was further considered interesting to determine whether expression levels of members of the inflammasome was affected by MDP stimulation.

## Materials and Methods

### Ethics Statement

The Scientific Ethics Committee of the Copenhagen Region approved the study. All patients gave their written informed consent before participation and the project fulfilled the Helsinki V Declaration.

### Patients and Genotyping of CARD15 Variants

18 patients with CD and 14 controls were included. Patients with CD were eligible for enrolment only if they had quiescent disease, and had not been administered any immunosuppressive drugs one month prior to sampling. Disease activity was evaluated by global assessment by the physician. Both CD patients and control subjects were screened for the common CARD15 variants: SNP8, SNP12, and SNP13 by the single strand conformation polymorphism (SSCP)[22]. PCR amplification was performed using primers for the polymorphic segments (exon 4e, exon 8, and exon 111, respectively). PCR products were subsequently separated on a SSCP gel. Subjects carrying any of the CARD15 variants were excluded. However, four patients homozygotic for the SNP8 CARD15 variant were included as disease controls in some experiments. CARD15 gene sequencing was not performed.

### Isolation, Culturing, and Stimulation of Human Monocytes

Venous blood was drawn in EDTA (10 mM) from the cubital vein. Peripheral mononuclear cells were isolated by density gradient centrifugation of blood diluted 1∶1 in pyrogen free saline over Ficoll-Paque (GE Healthcare Bio-sciences, Hillerød, Denmark). Monocytes were isolated by an indirect magnetic labelling method allowing isolation of untouched monocytes from human peripheral blood (Miltenyi Biotec, Bergish Gladbach, Germany). Non-monocytes were labelled using biotin conjugated antibodies against CD3, CD7, CD16, CD19, CD56, CD123, and glycophorin A, followed by indirect magnetic labelling by anti-biotin microbeads. The labelled cell suspension was transferred to a MACS LS separation column resulting in the separation of purified monocytes. Cells were kept at 4–8°C during the separation steps. Monocyte purity was assessed by flow-cytometry using fluorescence marked CD61, CD14, CD3, CD45 and IgG1 antibodies (Becton-Dickinson, Franklin Lakes, NJ, USA). Viability was measured by trypan dye exclusion. Isolated monocytes were kept in teflon wells (CCT-Europe, Ede, the Netherlands) and grown in a humid 5% CO_2_ atmosphere at 37°C at a cell density of 1×10^6^ cells per well. The monocytes were cultured over night in RPMI media (Invitrogen, Paisley, UK) supplemented with 10% heat inactivated human serum (Cambrex, Vallensbæk, Denmark), 1 mM pyrovate, 50 µg/ml gentamycin, 50 µg/ml penicillin and 50 µg/ml streptomycin. For stimulation, the media was replaced with Opti-Mem I Reduced Serum medium with antibiotics as mentioned above and highly purified MDP (50 ng/ml–5 µg/ml) (InvivoGen, San Diego, CA, USA). All materials were obtained from Invitrogen unless otherwise stated. Cells were harvested after 0, 4, and 24 hours.

### Monocyte Purity and Survival

The purity of monocytes was 91.4% (82.8–95.2). Viability after cell separation determined by dye exclusion was >99%. Viability remained high (>99%) after culture, and no differences were observed in cells stimulated by MDP between controls and CD patients.

### RT qPCR

RNA from stimulated monocytes was isolated by NucleoSpin columns (Macherey-Nagel, Düren, Germany). The amount of RNA was measured by spectrophotometry. The integrity and quality of the isolated RNA was determined by evaluation of RNA electrophoresis for each sample. cDNA was synthesised from 1–2 µg RNA by SuperScript III reverse transcriptase (Invitrogen, Paisley, UK).

RT qPCR analysis was performed using Brilliant SYBR Green QPCR Master Mix Kit and the PCR reaction was run on a Stratagene Mx3000P thermocycler (Stratagene, La Jolla, CA, USA). The primer sequences were taken from PrimerBank: IL-1β forward CTCGCCAGTGAAATGATGGCT, IL-1β reverse GTCGGAGATTCGTAGCTGGAT, TNF-α forward ATGAGCACTGAAAGCATGATCC, TNF-α reverse GAGGGCTGATTAGAGAGAGGTC, caspase 1 forward AGTGCAGGACAACCCAGCTA, caspase 1 reverse AGATAATGAGAGCAAGACGTGTG, NALP3 forward TCTCATGGATTGGTGAACAGC, NALP3 reverse GGTCCCCCAGAGAATTGTCA. The housekeeping gene Ribosomal Protein, Large, P0 (RPLP0) was used as a loading control: RPLP0 forward GCTTCCTGGAGGGTGTCC, RPLP0 reverse GGACTCGTTTGTACCCGTTG (MWG, Ebersberg, Germany). Relative expression of the mRNA was calculated from a reference sample taken from a pool of healthy donor monocytes stimulated for 4 hours.

### Immunoblotting

Monocytes were lysed with RP1 lysis buffer (Macherey-Nagel, Düren, Germany) and treated as according to the RNA-protein purification protocol. The protein flow through was resolved in RIPA buffer with 10% SDS. The samples were then heated at 95 °C for 5 minutes and run on a 4–12% gradient SDS acrylamaide gel under reducing conditions. Proteins were transferred to a Hypond ECL membrane (Amersham Biosciences, Freiburg, Germany). After blocking with 5% milk, the membranes were incubated with the appropriate primary antibodies overnight at 4°C and subsequently incubated with a horse radish peroxidase-conjugated secondary antibody (DakoCytomation, Copenhagen, Denmark) followed by ECL detection (Amersham). After blotting with antibodies against phospho-proteins or the active forms, the membranes were stripped and blotted with the unphosphorylated form, followed by blotting with GAPDH for the loading control. The primary antibodies were: IL-1β, active IL-1β, caspase 1, active caspase 1, NALP3, ASC, CARD8, p38, IKKα/β, JNK and GAPDH.

### ELISA

To determine activity of the inflammasome, secreted caspase 1 and IL-1β was investigated by ELISA technique (caspase 1 assay by R&D Systems, Minneapolis, MN, USA and IL-1β assay by Amersham, Buckinghamshire, UK).

### Statistics

Nonparametric statistics were applied. The Mann Whitney U test was used to compare medians. A significance level of 0.05 was applied.

## Results

### Effect of MDP on Cytokine Expression Levels

**Figure 1:**
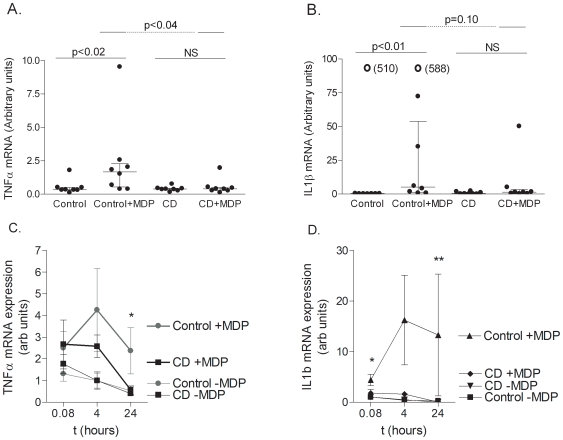
Kinetics of MDP-induced mRNA expression of TNF-α and IL-1β. RT-qPCR of mRNA of TNF-α (A) and IL-1β (B). Kinetic studies of mRNA-response (C and D). Isolated monocytes were stimulated for 5 minutes (0.08 hours), 4 hours and 24 hours. Stimulation with MDP increased the level of mRNA transcription in monocytes from control subjects (p<0.01 and p<0.02 for TNF-α and IL-1β, respectively), whereas an upregulation was not seen in monocytes isolated from CARD15 non-mutated CD patients. Arbitrary units. Due to high variability in IL1β expression in both control and CD patients, these expression data were normalised to expression values at t = 0 hours. *p<0.05; **p<0.01.

**Figure 2:**
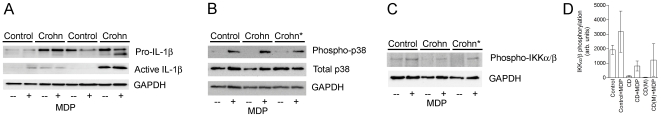
MDP-induced expression IL-1β and activation of p38 and IKKα/β. Immunoblotting of IL-1β (A), p38 (B), and IKKα/β (C). Quatitative IKKα/β phosphorylation, bars represent ranges (D). Monocytes were stimulated for 24 hours with MDP. IL-1β expression was increased in CD patients, but no response was seen to MDP. Monocytes stimulated with MDP had increased p38 phosphorylation, regardless of disease status. Contrary to the p38 response, reduced IKKα/β phosphorylation was seen in CD regardless of CARD15 status. Control monocytes did respond to MDP stimulation by increasing IKKα/β phosphorylation. CD: Crohn's disease. Crohn*: SNP8 homozygote.

To determine whether CARD15 stimulation resulted in expression of typical NFκB dependent pro-inflammatory cytokines, the mRNA expression levels of TNF-α and IL-1β were measured in monocytes from controls and from CD patients without disease linked CARD15 mutations. No differences in the mRNA expression levels were seen at baseline between CD patients and controls. Monocytes from control subjects had an increased TNF-α and IL-1β expression at the mRNA level after 24 hours of MDP stimulation (p<0.02 and p<0.01 respectively) (Fig. 1A and B). A similar increased expression was seen after 4 hours, but this did not reach statistical significiance when compared to stimulated cells from patients with CD (Fig. 1C and D). In contrast to this, CD patients had no increase in cytokine mRNA expression levels when stimulated with MDP. The expression level of TNF-α and IL-1β mRNA in MDP-stimulated control monocytes was higher than in MDP-stimulated CD cells (p<0.04 and p = 0.10, respectively). In contrast to the mRNA expression results, a constitutively higher IL-1β expression was found on the protein level in CD compared to control (Fig. 2A). MDP stimulation did not alter the protein expression of IL-1β in controls or CD patients.

### Effect of MDP on Expression of NALP3-Inflammasome Related Molecules

**Figure 3:**
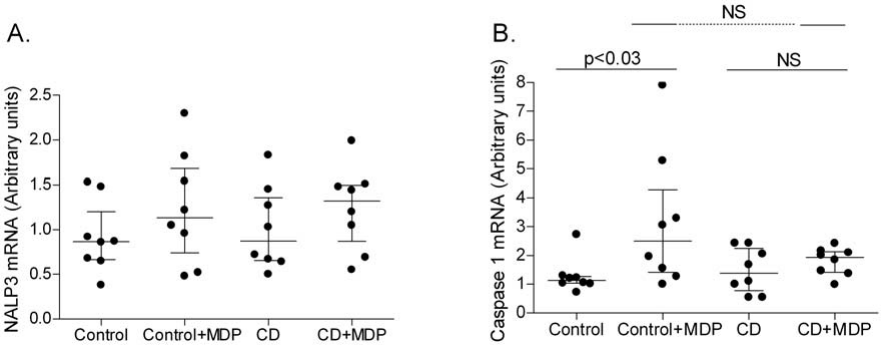
MDP-induced mRNA expression of NALP3 and caspase 1. RT-qPCR of mRNA of inflammasome member NALP3 (A) and caspase 1 (B). Arbitrary units. Isolated monocytes were stimulated for 24 hours. Stimulation with MDP increased the level of caspase 1 expression in control cells (p<0.03), whereas such an increase was not found in CD monocytes.

Expression of NALP3 mRNA was equal in CD and control monocytes. The mRNA level was not altered by MDP stimulation (Fig. 3A). Contrary to this, MDP stimulation clearly resulted in increased caspase 1 expression in control monocytes, whereas a similar effect was not seen in CD monocytes (p<0.03, Fig. 3B). Basal caspase 1 expression was equal in control and CD monocytes.

### Analysis of Activation of Activation Pathways Downstream of CARD15

Expression and phosphorylation of the p38 MAP kinase pathway was subsequently investigated, as a marker to determine if differences in downstream activation after CARD15 could explain the decreased response to MDP in CD. There was no detectable phospho-p38 in unstimulated controls and CD patients with or without CD associated CARD15 variants (Fig. 2B). Stimulation with MDP highly activated the p38 MAPK as seen by increased amounts of phospho-p38. Interestingly, this increase was found in both control and CD monocytes regardless of CARD15 status. Thus no major differences were found between control and CD in activation of the p38 pathway. Contrary to CD monocytes, control monocytes had an increased IKKα/β phosphorylation without stimulation. The IKKα/β phosphorylation increased substantially in MDP stimulated control monocytes compared to CD monocytes, which had low or no detectable IKKα/β phosphorylation upon MDP stimulation (Fig. 2C). No significant differences were seen between CD monocytes regardless of CARD15 variant status. This suggests that lack of NFκB activation by MDP in CD could explain the decreased response to MDP.

### Activation of the Inflammasome and IL-1β Maturation

**Figure 4:**
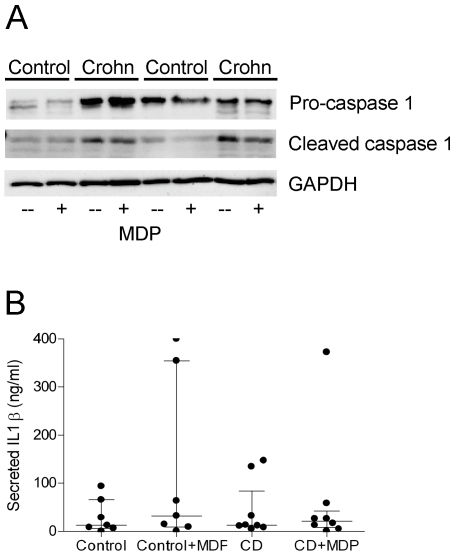
Caspase 1 processing and IL-1β release. Immunoblotting of caspase 1 (A). CD patients had increased basal expression of pro-caspase 1 and active cleaved caspase 1. No response was seen to MDP stimulation. ELISA measurement of released active IL-1β (B). Media was taken from monocytes stimulated for 24 hours with MDP. No differences in IL-1β secretion were detected.

**Figure 5:**
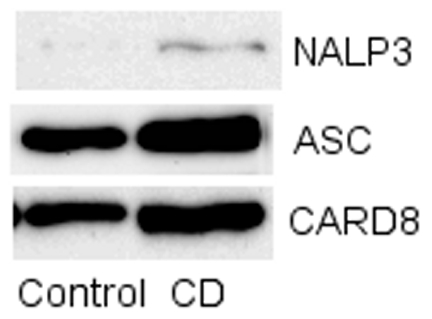
Expression of inflammasome related proteins. Immunoblotting of NALP3, ASC, and CARD8. Monocytes expressed all these proteins involved in caspase 1 maturation.

Caspase 1 protein was constitutively highly expressed in CD monocytes, and was cleaved to its active form in unstimulated cells (Fig. 4A). No increases in the expression level or cleavage of caspase 1 were seen upon stimulation with MDP neither in CD nor in controls. Monocytes expressed the proteins of the NALP3-inflammasome on the protein level: NALP3, ASC, and CARD8 (Fig. 5). Released activated caspase 1 to the media was, however, equal in CD and control (data not shown). As described earlier the basal level of pro-IL-1β was increased in CD compared to controls. The amount of matured IL-1β was also increased in CD, but in all cases IL-1β protein expression was independent of MDP stimulation. The release of mature IL-1β was also independent on disease stage and MDP stimulation and equal in CD and control monocytes (Fig. 4B). This suggests that the inflammasome is constitutively active in CD, but that the inflammasome activity is not dependent on MDP stimulation in human monocytes, neither in controls, nor in CD. The constitutively active caspase 1 found in CD explain the increased expression of mature IL-1β in CD compared to control monocytes.

## Discussion

We describe an impaired response to MDP in monocytes from CD patients without disease linked CARD15 variants. The dysfunctional response is in accordance with the increasing evidence of an impaired innate immune function in CD, and in particular monocytes isolated from CD patients [1], [23], [24]. Upregulation of proinflammatory NFκB and MAP kinase dependent cytokine gene transcription is the classic response to activation of CARD15-dependent pathways [5], [6]. It is interesting to investigate a response to MDP, since this is the most specific stimulus of CARD15 dependent pathways [5]. Decreased or absent activation of CARD15 dependent pathways has previously been described for three CARD15 variants associated with CD [5], [16], [19], [25], and the response was shown to be dependent on one specific variant (SNP 13 (1007fs) homozygote) causing less activation than the other two [14]. These studies have primarily shown a decreased chemokine secretion in CD associated CARD15 variants stimulated with MDP alone, whereas IL-1β and TNF-α secretion was unaffected or not investigated. Non-mutated CARD15 CD cells responded normally in terms of cytokine secretion when co-stimulated with various PAMPs, but no cytokine production was seen in cells only stimulated with MDP in neither CD, nor control monocytes [19]. It has previously been reported that MDP-dependent TNF-α secretion requires co-stimulation with LPS or other PGN, whereas TNF-α mRNA is greatly increased by MDP stimulation alone, but not translated into TNF-α protein [26]. This is most likely the explanation for the lack of increase in IL-1β protein expression and release seen in our experiments compared to the above sited studies. Thus, we were able to induce secretion of IL-1β with co-stimulation of monocytes with LPS and MDP (data not shown).

Our results suggest that MDP regulated pathways are inhibited in CARD15 non-mutated CD monocytes. It is possible that this lack of response might be overruled by co-activation of other LRR pathways, as some earlier published reports may indicate [16], [25], but it defines a unique response similar to CARD15 mutated responses. Subsequent analysis of CARD15 dependent pathways suggested that this difference was due to an impaired activation of the classical NFκB pathway in both non-mutated and mutated CARD15 monocytes, since MDP resulted in an impaired IKKα/β-phosphorylation, the initial step in the activation of the NFκB pathway. Interestingly, the monocytes from controls, CARD15 non-mutated CD patients, and CARD15 mutated CD patients induced p38 phosphorylation in response to MDP, whereas no detectable activation was seen on the JNK pathway. This pattern of activation of MAPK pathways is similar to what previously have been found in whole intestinal biopsies from patients with active IBD [27]. Although the response seems to be normal, it is interesting that it is seen in CARD15 mutated monocytes here.

The finding suggests that MDP dependent activation of p38 occurs through other MDP sensing PRRs in human monocytes. It is, however, also reassuring that p38 is activated by MDP in CD monocytes, as it confirms that control and CD cells have been stimulated. It is unlikely that the stimulation could be due to TLR2 dependent signalling, since MDP alone has not been shown to activate TLR2 [28]. The results reveal an impairment of a specific NLR pathway in CD monocytes not carrying the disease associated CARD15 variants, and therefore add evidence to the theory of a dysfunctional innate immune response in CD monocytes. It also emphasises that studies into the role of the NLR pathway in CD monocytes should employ CARD15 non-mutated CD monocytes as well as CARD15 non-mutated control monocytes, since non-mutated CARD15 monocytes from CD patients also appear to have dysfunctional PRR pathways. From animal experimental studies there is increasing evidence of how MDP-signalling seem to protect from inflammatory bowel disease. Chronic MDP-stimulation thus seem to downregulate signalling through other PRRs and protect mice from colitis [29], [30]. Whether similar effects are to be found in human remain to be investigated.

In order to map the other possible consequences of MDP stimulation of monocytes, the present study examined the effect of MDP on inflammasome molecule expression and activation. It was shown that caspase 1 expression on the mRNA level is positively regulated by MDP stimulation, whereas NALP3 expression is unaltered. This has not previously been described. Interestingly, this caspase 1-mRNA upregulation was inhibited in CD CARD15 non-mutated monocytes, which further substantiates a decreased MDP response in CD monocytes. In this experimental setting MDP did not activate the inflammasome as evidenced by a lack of caspase 1 cleavage. Thus, we found a constitutive activation of the inflammasome in CD monocytes regardless of the CARD15 status. This might contribute to the pro-inflammatory state found in CD, and may suggest that CARD15 acting as a scaffold protein for caspase 1 activation remains intact. Whereas a similar pattern has previously been described based on immunohistochemical double staining studies [31], activation of the inflammasome in quiescent disease stages has not earlier been reported. The constitutively active inflammasome/caspase 1 could explain the higher levels of maturated IL-1β found in CD in this study.

In conclusion, this study shows that MDP-dependent pathways are inhibited in CD monocytes without the disease associated CARD15 variants, which indicates that the innate immune response is deficient in CD. We found no evidence of a direct activation of the inflammasome by MDP in human monocytes, but an upregulation on the mRNA level of members of the cytokine processing system, which was also impaired in CD. The results indicate that future therapies aimed at restoring CARD15 signalling might be effective regardless of CARD15 status.
